# Engineering the L-Arabinose Isomerase from *Lactobacillus sakei* for Whole-Cell Conversion Production of D-Tagatose Under High Substrate Concentration Conditions

**DOI:** 10.3390/molecules31162756

**Published:** 2026-08-08

**Authors:** Ren He, Wei Liu, Bingyi Tao, Shaoxiong Liang, Xuchong Tang

**Affiliations:** 1 Academy of Advanced Carbon Conversion Technology, Huaqiao University, Xiamen 361021, China; 35536@hqu.edu.cn (R.H.);; 2 College of Chemical Engineering, Huaqiao University, Xiamen 361021, China; 3 BaYeCao Health Industry Research Institute Co., Ltd., Xiamen 361021, China

**Keywords:** L-arabinose isomerase, D-tagatose, site-directed mutagenesis, whole-cell bioconversion

## Abstract

D-tagatose, a low-calorie sugar substitute, widely used in the food and pharmaceutical industries, can be produced from the substrate D-galactose using L-arabinose isomerase (L-AI). L-AI genes derived from *Lactobacillus fermentum* (LFAI), *Lactobacillus brevis* (LBAI), *Lactobacillus plantarum* (LPAI), and *Lactobacillus sakei* (LSAI) for the bioconversion of D-galactose into D-tagatose using whole *Escherichia coli* (*E. coli*) cells were investigated. The temperature, time, pH, whole-cell concentration, and metal ion concentration were optimized to enhance catalytic production. The results revealed that at the low substrate concentration of 10 g/L, LFAI, LBAI, LPAI, and LSAI exhibited considerable catalytic activity, with conversion rates of 55.31%, 68.17%, 61.45%, and 58.22%, respectively. In contrast, at the high substrate concentration of 250 g/L, their conversion rates were 19.78%, 18.47%, 20.26%, and 36.24%, respectively, with LSAI demonstrating superior catalytic performance. In order to facilitate the industrial production of D-tagatose, site-directed mutagenesis was performed on LSAI. Among the engineered LSAI variants, the P99H, K142R, and L465R mutants all showed increased catalytic rates for D-tagatose. Specifically, at the high substrate concentration of 250 g/L, the P99H mutant achieved a conversion rate of 45.73%, representing a 32.7% improvement compared to the optimized LSAI. These results indicate that the designed site-directed mutagenesis strategy effectively enhanced whole-cell bioconversion performance of LSAI toward D-galactose.

## 1. Introduction

D-tagatose is a rare natural ketohexose, which is an epimer of D-fructose at C-4 and a keto-isomer of D-galactose [1,2]. It is a low-calorie functional sweetener that can replace traditional high-calorie sweeteners, with uses in food and medical applications [3,4]. Furthermore, D-tagatose possesses numerous important physiological functions, such as promoting blood health [5], preventing dental caries [6], improving intestinal flora [7], and controlling obesity [8]. The U.S. Food and Drug Administration (FDA) has listed D-tagatose in the Generally Recognized as Safe (GRAS) food list since 2001 [9]. On 14 December 2005, the European Union (EU) formally approved tagatose as a “novel food ingredient,” without any limitations on its application [10].

Currently, the main methods for producing D-tagatose are chemical synthesis and biosynthesis [11]. The chemical method employs expensive catalysts and generates by-products, which complicates the purification process and renders it unsuitable for industrial production [12]. In comparison, the biosynthetic approach for tagatose synthesis offers the advantages of mild reaction conditions, a clean and low-carbon footprint, and high catalytic specificity [13], rendering it highly competitive for the production of D-tagatose. L-arabinose isomerase is an intracellular enzyme that catalyzes the reversible isomerization of D-galactose to D-tagatose and of L-arabinose to L-ribulose [14,15,16]. Several L-AIs have been successfully characterized to date from mesophilic, thermophilic, and hyperthermophilic bacteria [13,17,18,19,20,21,22,23,24].

Rational design has been widely applied to modulate the functional and structural properties of proteins. In contrast to rational design, irrational design utilizing directed evolution offers the possibility of modifying proteins in the absence of precise structural and biochemical information. The most commonly used method for directed evolution is error-prone polymerase chain reaction (EP-PCR). For example, using EP-PCR technology, L-AI from *Geobacillus stearothermophilus* was mutated. The resulting mutants showed significantly increased isomerization activity toward D-galactose, improved catalytic parameters, an optimized reaction temperature, and a markedly higher D-tagatose yield [25]. However, the individual mutant screening technique is both time consuming and labor-intensive. Mutating sites near the enzyme’s active site or substrate-binding region can effectively address this issue. Site-directed mutagenesis was performed on L-AI from *Geobacillus stearothermophilus*, mutating His at position 118 to Thr, resulting in mutant exhibited higher affinity for D-galactose. Compared with the wild type, the enzyme activity was increased by 1.8-fold and the catalytic activity was increased by 2.3-fold [26]. Similarly, L-AI from *Lactobacillus fermentum* was investigated. Mutations were introduced at three sites: Asp268, Asp269, and Asp299. The resulting mutant strains D268K, D269K, and D299K not only improved conversion rates of D-tagatose from D-galactose substrate at pH 5.0 in a borate-buffered reaction system, but also enhanced pH stability [27].

In this study, L-AIs from *Lactobacillus fermentum* (LFAI), *Lactobacillus brevis* (LBAI), *Lactobacillus plantarum* (LPAI), and *Lactobacillus sakei* (LSAI) were selected. The encoding gene *araA* from these strains was cloned and overexpressed in recombinant *E. coli* BL21 (DE3) to construct *E. coli* engineered strains. The optimal reaction conditions for catalytic production of D-tagatose were investigated, and LSAI was identified as the best D-tagatose production invertase for whole-cell catalysis at high substrate concentrations. We performed site-directed mutagenesis on the L-AI gene of LSAI, mutating proline at position 99 to histidine (P99H), lysine at position 142 to arginine (K142R), and leucine at position 465 to arginine (L465R).

## 2. Results and Discussion

### 2.1. Construction of Recombinant Plasmid

**Figure 1:**
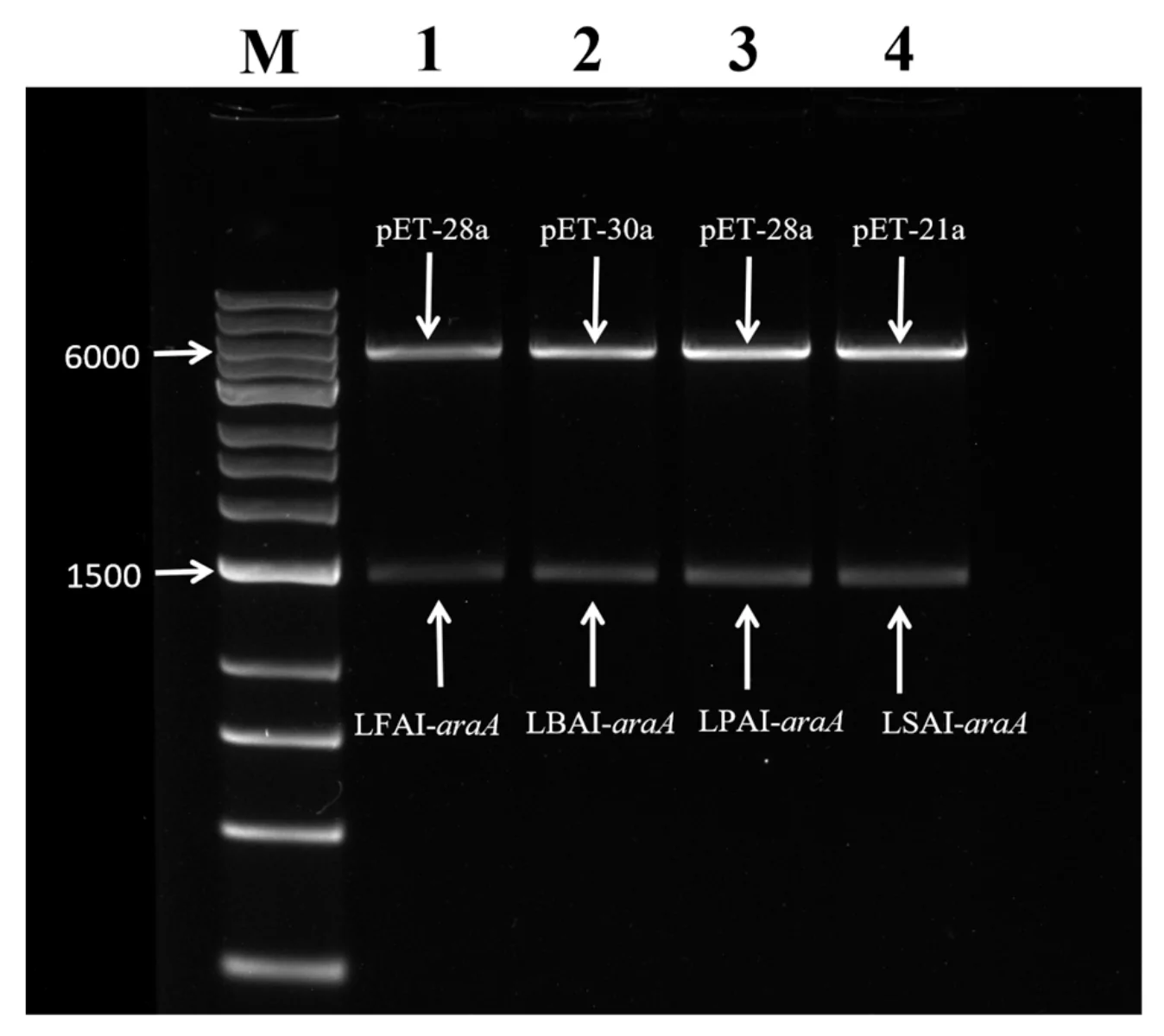
Dual enzyme digestion validation of four recombinant plasmids. Notes: Lane M: DNA marker, Lane 1: pET-28a-LFAI-*araA*/EcoR I and Xho I, Lane 2: pET-30a-LBAI-*araA*/EcoR I and BamH I, Lane 3: pET-28a-LPAI-*araA*/EcoR I and Xho I, Lane 4: pET-21a-LSAI-*araA*/Nde I and Hind III.

Recombinant plasmids individually transformed into *E. coli* DH5a were extracted for double enzyme digestion verification. Band sizes were consistent with the expectation, indicating the initial successful construction of the four recombinant plasmids (Figure 1). Finally, the recombinant plasmids were sent to Genewiz (Suzhou, China) for sequencing, which confirmed the correct insertion of the *araA* gene into each corresponding plasmid.

### 2.2. Optimization of Target Protein Expression

The growth of strains was measured in LB medium without inducer (Figure S1). Based on the growth curves, we compared the protein expression levels of four recombinant strains during the early, middle, and late logarithmic growth and the stationary phases. It was found that the exponential growth phase of all recombinant strains was 6–10 h. During this period, the strains grew rapidly with active intracellular metabolism and stable physiological characteristics, making it the optimal period for inducing heterologous protein expression. Therefore, the time within this range was selected for the subsequent optimization of induction conditions.

**Figure 2:**
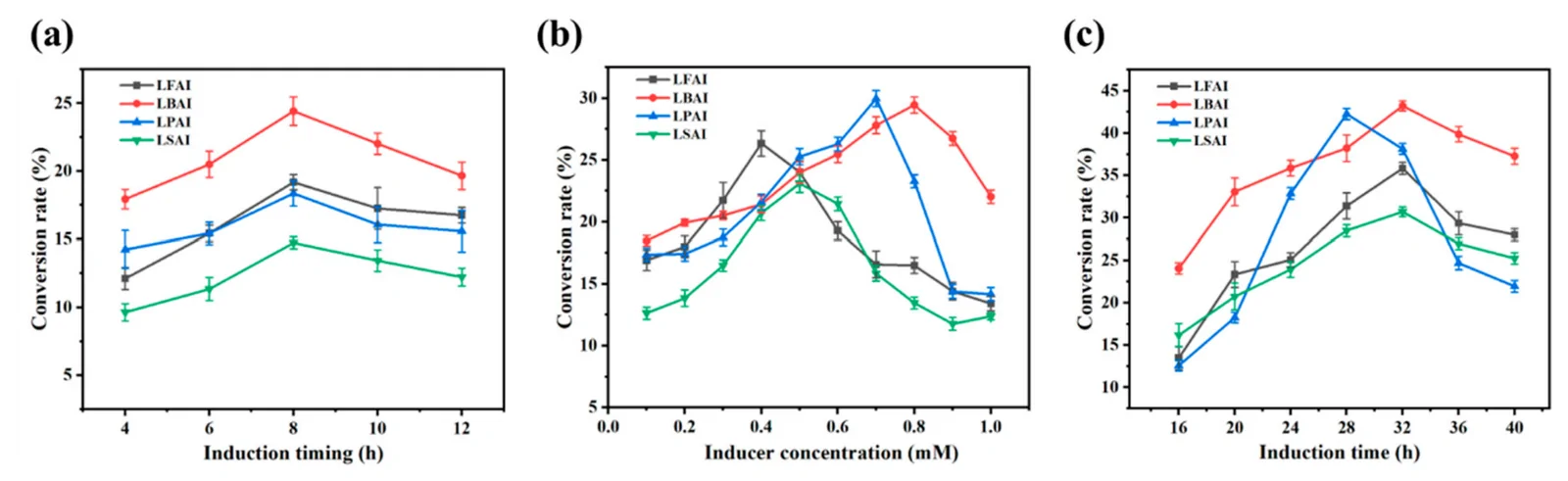
(**a**) Effect of inducer addition timing on conversion rate; (**b**) effect of inducer concentration on the conversion rate; (**c**) effect of induction time on conversion rate. (*n* = 3 biological replicates).

Induction timing, duration, and inducer concentration affect conversion rates (Figure 2). According to the growth curve, the inducer addition timing in this study was set between 4 and 12 h. When IPTG was added for induction at 8 h of culture, the catalytic performance of L-AI was the best (Figure 2a). At this time, the OD_600_ was in the range of 0.6–0.8, and the strains were in the middle of the logarithmic growth phase, with strong growth and expression capabilities, which was the optimal period for induction. The final concentrations of the inducer tested ranged from 0.1 to 1.0 mM and the optimal final IPTG concentrations for LFAI, LBAI, LPAI, and LSAI were 0.4 mM, 0.8 mM, 0.7 mM, and 0.5 mM, respectively (Figure 2b). On the basis of the optimization results above, induction was performed at 16 °C for 16–40 h. With the increase in induction duration, the conversion rate first increased and then decreased, and the optimal induction durations for LFAI, LBAI, LPAI, and LSAI were determined to be 32 h, 32 h, 28 h, and 32 h, respectively (Figure 2c).

### 2.3. Optimization of Whole-Cell Production of D-Tagatose

**Figure 3:**
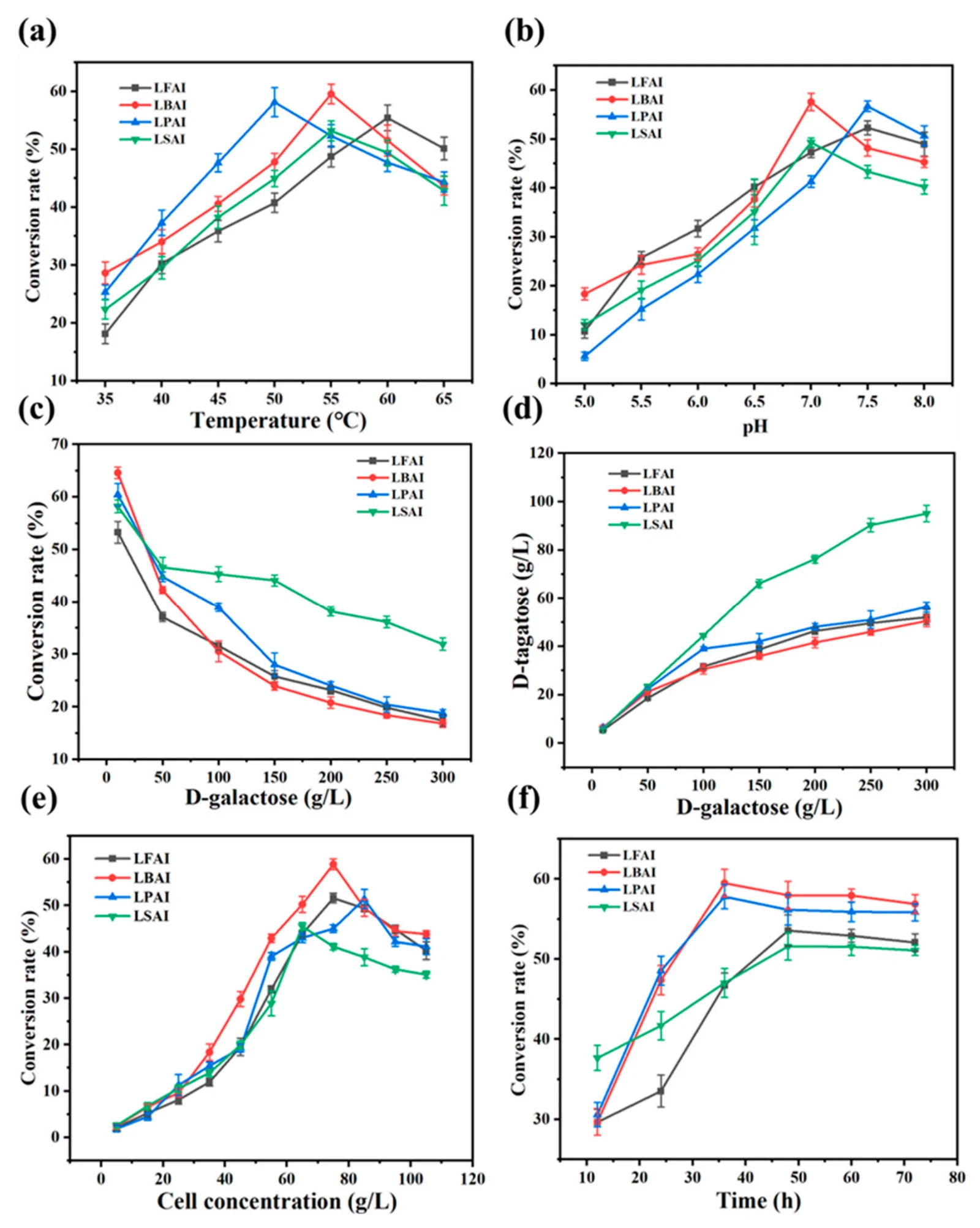
Single factor optimization of conversion of D-galactose into D-tagatose. (**a**) Reaction temperature; (**b**) initial pH; (**c**,**d**) substrate concentration; (**e**) cell concentration; (**f**) reaction time. (*n* = 3 biological replicates).

**Figure 4:**
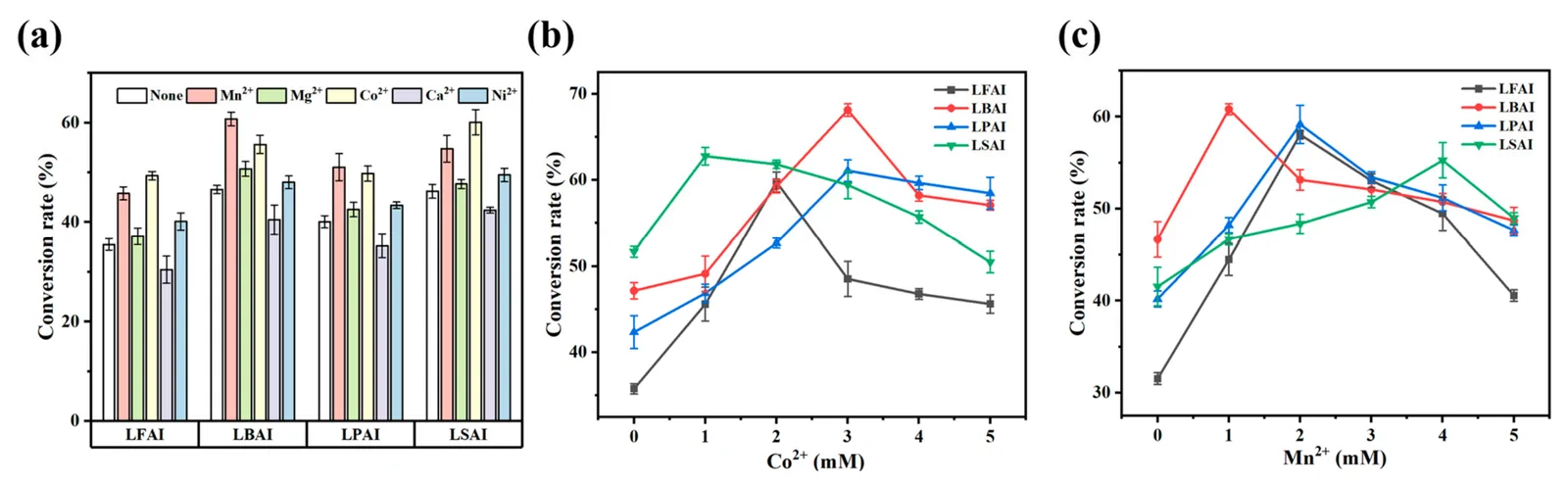
(**a**) Effect of metal ions on L-AI activity; (**b**) effect of different concentrations of Co^2+^ on the conversion rate; (**c**) effect of different concentrations of Mn^2+^ on the conversion rate. (*n* = 3 biological replicates).

Temperature, time, pH, cell concentration, substrate concentration, and metal ions affected the whole-cell bioconversion performance (Figure 3 and Figure 4). The conversion rate of D-tagatose initially increased then decreased with rising temperature (Figure 3a). This is because increased thermal motion of enzyme molecules at higher temperatures enhances the binding efficiency between the enzyme and substrate. However, beyond a certain temperature, the integrity of cell membranes is damaged, which hinders the transmembrane transport of substrates and products, weakens the catalytic activity of enzymes, and ultimately leads to a decrease in conversion rate [28]. The optimal whole-cell catalytic temperatures were determined to be 60 °C for LFAI, 55 °C for LBAI and LSAI, and 50 °C for LPAI. The optimal pH for D-tagatose biotransformation from D-galactose was 7.5 for LFAI and LPAI, and 7.0 for LBAI and LSAI (Figure 3b). In overly acidic or alkaline environments, the cellular structure of the bacteria is disrupted, inhibiting bacterial growth and significantly decreasing L-AI activity. In contrast, in a neutral environment, the bacteria can maintain a stable physiological metabolic state, and L-AI can also retain its optimal catalytic activity, maximizing the conversion rate [29]. The product yield varied depending on the D-galactose concentration. As the substrate concentration increased, the conversion rate declined (Figure 3c), whereas the D-tagatose titer increased (Figure 3d). At a D-galactose concentration of 250 g/L, the yields of D-tagatose catalyzed by LFAI, LBAI, LPAI, and LSAI were 49.52, 45.91, 50.93, and 90.24 g/L, respectively. Since further increases in substrate concentration did not significantly improve the yield, 250 g/L was selected as the optimal substrate concentration. The optimal whole-cell concentrations for maximum D-tagatose conversion by LFAI, LBAI, LPAI, and LSAI were determined to be 75, 75, 85, and 65 g/L, respectively, beyond which further increases led to a reduction in D-tagatose synthesis (Figure 3e). This effect may be attributed to the diffusion inhibition of enzymes and substrates at higher cell concentrations, resulting in decreased mass transfer efficiency [30]. The conversion rate of D-tagatose remained stable after 48 h for LFAI and LSAI, and after 36 h for LBAI and LPAI, with no significant changes up to 80 h (Figure 3f). Thus, the optimal reaction times were 48 h for LFAI/LSAI and 36 h for LBAI/LPAI, and extending the reaction time further did not affect the product yield.

Most reported L-arabinose isomerases are metalloproteins, and they exhibit optimal enzymatic activity only when metal ions are bound to their active sites [31]. Figure 4a shows that Mn^2+^, Mg^2+^, Co^2+^, and Ni^2+^ can all enhance the activity of L-AI to a certain extent, with Mn^2+^ and Co^2+^ demonstrating the most significant enhancement. Possibly because Mn^2+^ and Co^2+^ have appropriate ionic radii and high charge densities, they facilitate stable coordination with amino acid residues in the enzyme’s active center, thereby enhancing whole-cell bioconversion performance [32]. Although Mg^2+^ and Ni^2+^ can also improve activity through a similar mechanism, their weaker coordination capacity results in a less pronounced effect. Ca^2+^ inhibits L-AI activity, possibly due to its larger ionic radius and lower charge density, which may distort the binding mode at the active site and destabilize the enzyme–substrate complex. At 1 mM, Mn^2+^ and Co^2+^ showed comparable catalytic abilities. Further studies at 0–5 mM (Figure 4b,c) revealed that low concentrations of Mn^2+^ and Co^2+^ promoted L-AI enzyme activity, while high concentrations inhibited it, with Co^2+^ being more effective than Mn^2+^. Under the catalysis of Co^2+^, the highest conversion rates of LFAI, LBAI, LPAI, and LSAI reached 59.78%, 68.12%, 62.77%, and 61.09%, respectively. Therefore, Co^2+^ was selected for subsequent reactions, with optimal concentrations of 2 mM, 3 mM, 3 mM, and 1 mM for LFAI, LBAI, LPAI, and LSAI, respectively.

### 2.4. Production of D-Tagatose Under Optimal Conditions

**Figure 5:**
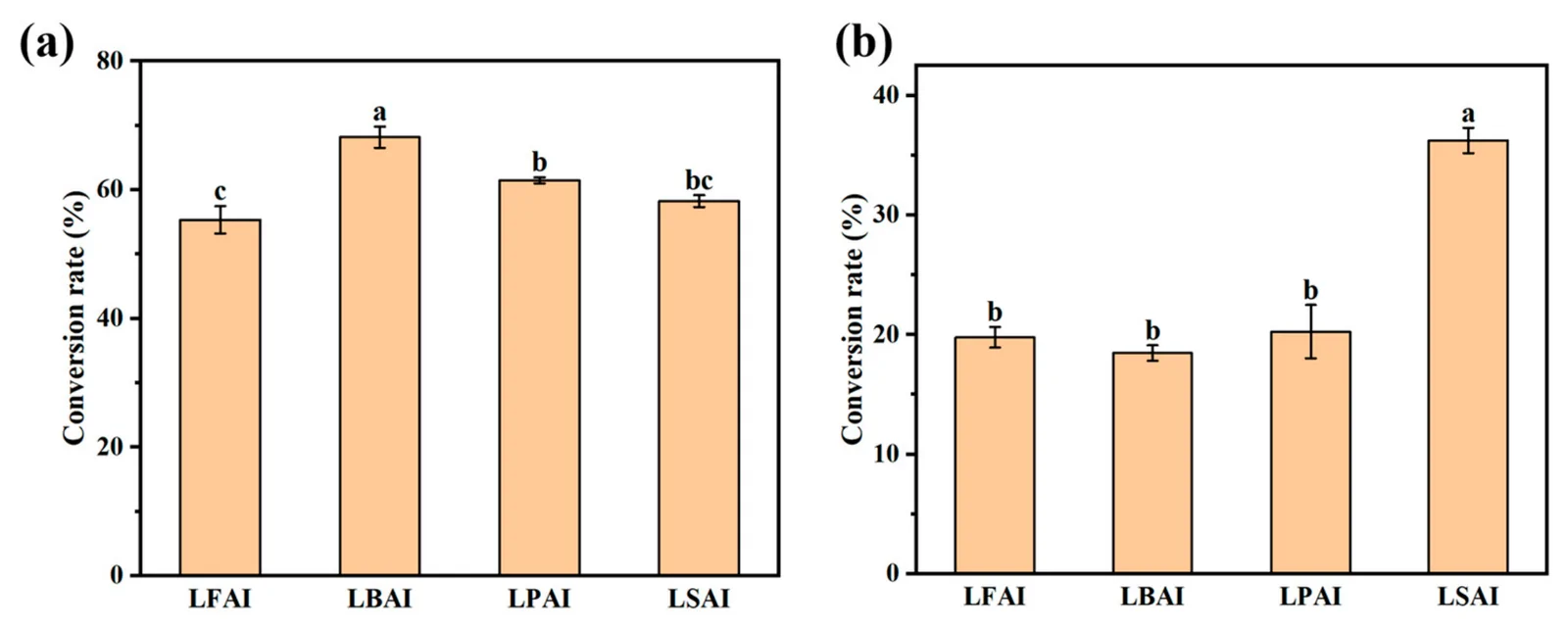
Results under optimal catalytic conditions: (**a**) 10 g/L; (**b**) 250 g/L. Different letters above the bars indicate significant differences among groups according to one-way ANOVA followed by Tukey’s multiple comparison test (*p* < 0.05). Data are presented as mean ± SD (*n* = 3 biological replicates).

Compared to in vitro enzymatic methods, whole-cell catalysis eliminates the need for cell disruption or enzyme purification and separation. The cell membrane protects enzymes from damage through shear forces [33], enhancing enzyme stability and reducing production costs. From both industrial and economic perspectives, whole-cell catalysis is more advantageous for the large-scale synthesis of D-tagatose [34]. *E. coli* has a strong ability to carry various heterologous genes, making it an ideal host for developing whole-cell catalysts [35]. Recombinant strains harvested at different culture times were used as whole-cell catalysts for D-tagatose production under optimal catalytic conditions. When the D-galactose concentration was 10 g/L, the conversion rates for LFAI, LBAI, LPAI, and LSAI were 55.31%, 68.17%, 61.45%, and 58.22%, respectively (Figure 5a). At a D-galactose concentration of 250 g/L, the conversion rates were 19.78%, 18.47%, 20.26%, and 36.24% for LFAI, LBAI, LPAI, and LSAI, respectively (Figure 5b). At low substrate concentrations, LBAI exhibited the highest activity, with LSAI showing slightly lower catalytic activity. At high substrate concentrations, LSAI exhibited a better catalytic effect with a conversion rate of 36.24%, while the conversion rates of LFAI, LBAI, and LPAI were all around 20%. Therefore, subsequent molecular modifications of LSAI were conducted to further improve D-tagatose yield.

### 2.5. Expression and Bioconversion Efficiency of Mutants

**Figure 6:**
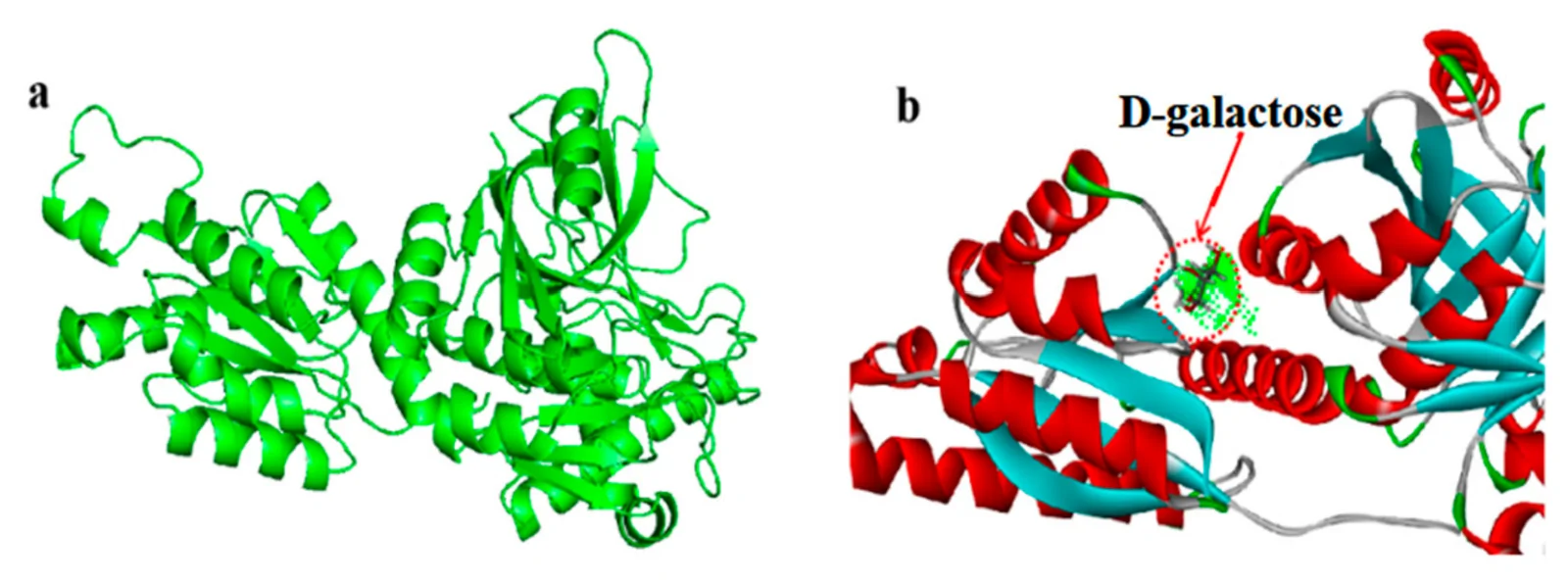
(**a**) Three-dimensional structure of LSAI; (**b**) molecular docking.

Homology alignment of the wild-type LSAI-*araA* sequence was performed using SWISS-MODEL (https://swissmodel.expasy.org/) (Table S1), and the three-dimensional (3D) structure of LSAI was constructed via homology modeling based on the optimal template (Figure 6a). Subsequently, molecular docking between the LSAI structure and the substrate D-galactose was simulated using Discovery Studio 2021 Client (Figure 6b). Candidate residues were identified based on their spatial relationship with the substrate-binding pocket and were subsequently subjected to single-point mutation scanning. For the mutations of proline 99 to histidine (P99H), lysine 142 to arginine (K142R), and leucine 465 to arginine (L465R), the predicted changes in binding free energy (ΔΔG_bind) were −0.69, −0.83, and −0.61 kcal/mol, respectively. These effects were classified as “STABILIZING” (Figure S2), indicating that the mutations may enhance the stability of the enzyme–substrate complex and improve substrate binding. Next, AlphaFold (https://alphafoldserver.com/) was employed to assess the quality of protein structure predictions for both the wild-type and the mutants (P99H, K142R, and L465R). The average pLDDT scores of the wild-type and the three mutants were 96.52, 96.29, 96.40, and 96.60, respectively, indicating that all predicted structures were of high confidence. Therefore, the P99H, K142R, and L465R mutants were selected for further study.

**Figure 7:**
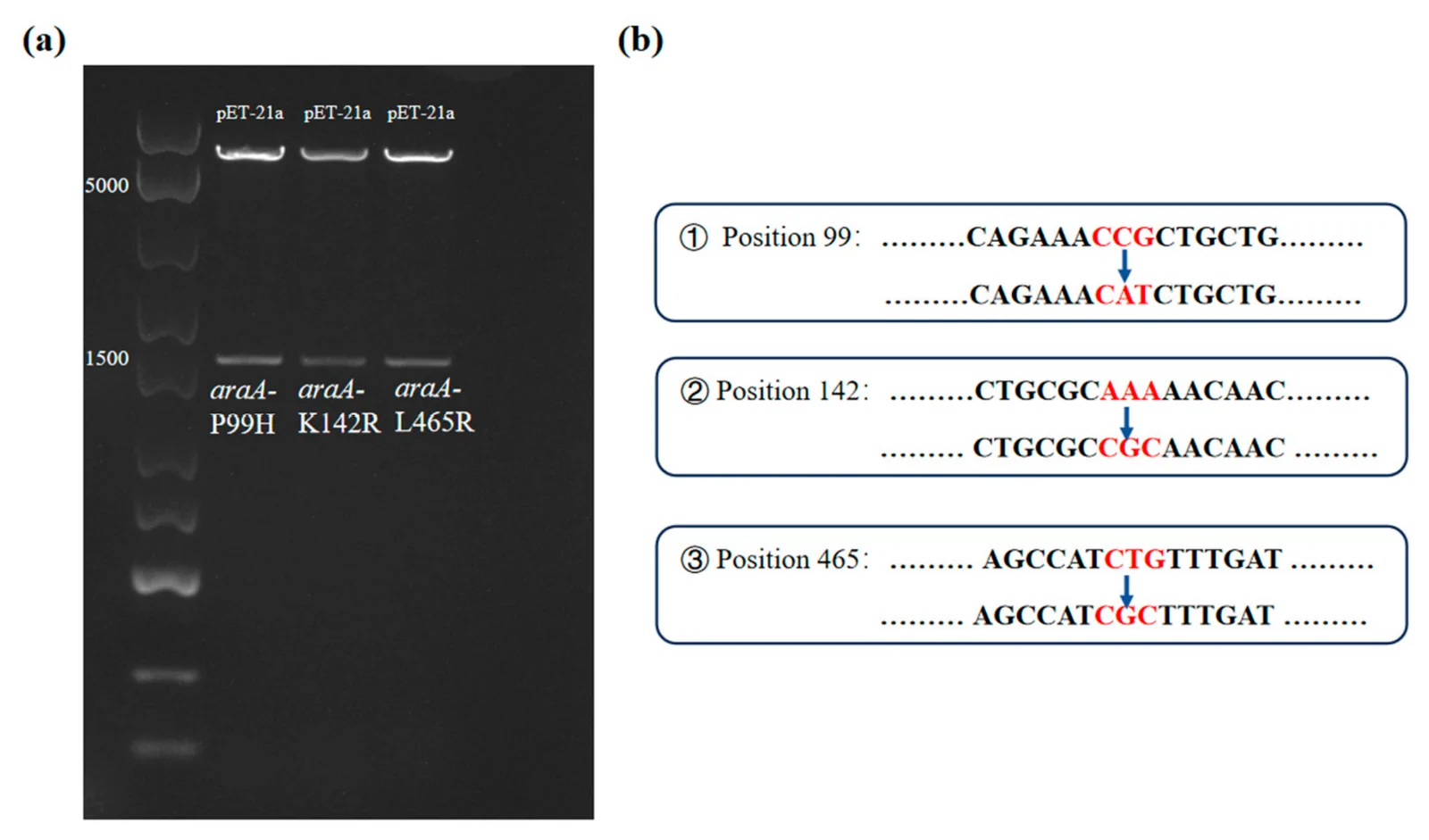
(**a**) Validation of double digestion of plasmids; (**b**) sequencing validation.

**Figure 8:**
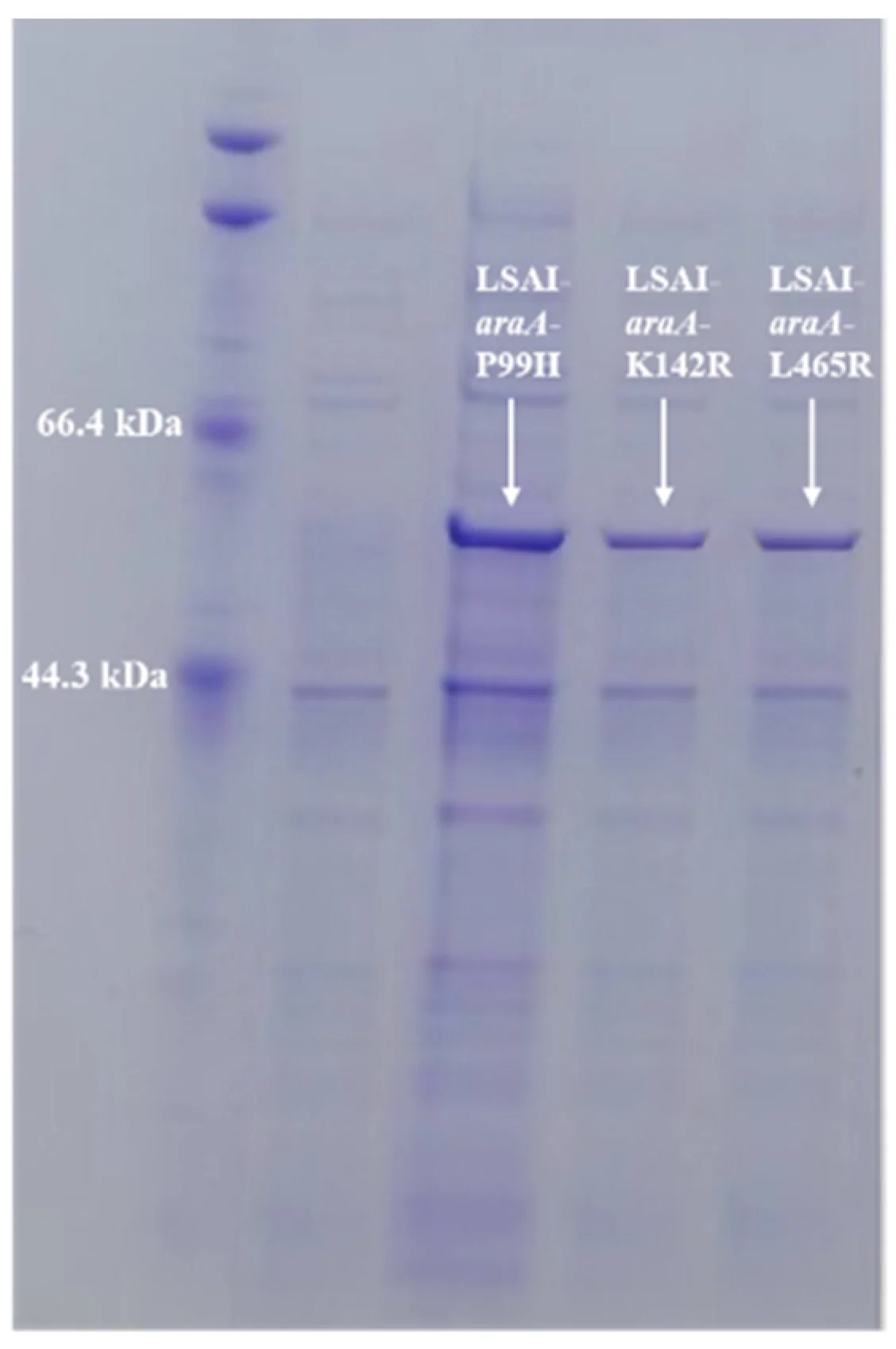
SDS-PAGE electrophoretic analysis of LSAI expressed by recombinant.

The mutant plasmid was verified by double digestion with Nde I and Hind III, yielding bands consistent with the expected sizes (Figure 7a). Subsequently, sequencing of the mutants confirmed the successful mutation at three sites (Figure 7b). The three mutant constructs were then transformed into *E. coli* BL21(DE3) to obtain mutant strains. After induction with IPTG, the recombinant strains were analyzed by SDS-PAGE electrophoresis (Figure 8). Using non-induced recombinant strains as controls, a distinct protein band around 56 kDa was observed in the IPTG-induced samples, consistent with the theoretical size of LSAI. This indicates that the genes of the three mutant proteins were all successfully expressed in *E. coli* BL21(DE3).

**Figure 9:**
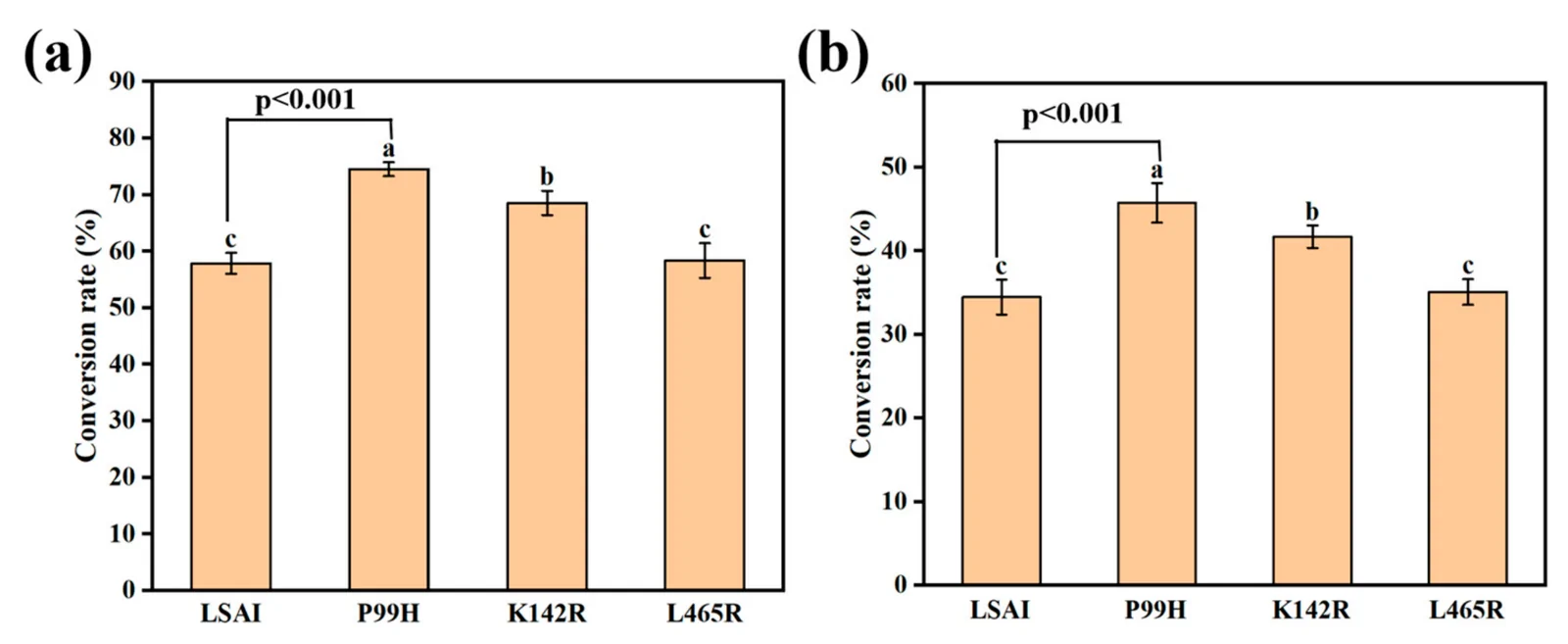
Conversion rate of mutants to produce D-tagatose: (**a**) 10 g/L; (**b**) 250 g/L. Different letters above the bars indicate significant differences among groups according to one-way ANOVA followed by Tukey’s multiple comparison test (*p* < 0.05). Data are presented as mean ± SD (*n* = 3 biological replicates). Notes: P99H is LSAI-*araA*-P99H, K142R is LSAI-*araA*-K142R, and L465R is LSAI-*araA*-L465R.

After induction and expression of the mutant strains, the cells were harvested by centrifugation and resuspended in PBS buffer (pH 7.0) containing 1 mM Co^2+^, and incubated with 10 g/L or 250 g/L D-galactose at 55 °C and 220 rpm for 48 h. The conversion rates of D-tagatose were measured to compare the bioconversion performance of LSAI-*araA*-P99H, LSAI-*araA*-K142R, and LSAI-*araA*-L465R (Figure 9). The results indicated that at the low substrate concentration of 10 g/L (Figure 9a), the conversion rate of D-tagatose catalyzed by LSAI was 57.86%. The conversion rate for the mutant LSAI-*araA*-P99H reached 74.53%, representing a 28.81% increase; for LSAI-*araA*-K142R, it was 68.55%, a 18.48% increase; while for LSAI-*araA*-L465R, it was 58.36%, with no significant improvement. At the high substrate concentration of 250 g/L (Figure 9b), the conversion rate of D-tagatose catalyzed by LSAI was 34.46%; the conversion rate of LSAI-*araA*-P99H was 45.73%, with a 32.7% increase in catalytic ability; that of LSAI-*araA*-K142R was 41.69%, an increase of 20.98%; similarly, the conversion rate of LSAI-*araA*-L465R was 35.08%, showing no significant improvement in catalytic ability. This may be because the amino acid substitution at the L465 site failed to effectively optimize the enzyme’s catalytic activity and thus could not promote D-tagatose production, suggesting that this site is not critical for the enzyme-catalyzed production of D-tagatose. In summary, the mutants LSAI-*araA*-P99H and LSAI-*araA*-K142R both improved D-tagatose production capacity, whereas LSAI-*araA*-L465R showed no significant enhancement, with LSAI-*araA*-P99H demonstrating the greatest improvement. A comparison of the D-tagatose conversion efficiency in this work with that reported in the literature (Table 1) shows that *L. sakei*-*araA*-P99H has obvious advantages in whole-cell catalytic production of D-tagatose under high substrate concentration conditions [28,31,32,34,36,37,38,39].

## 3. Materials and Methods

### 3.1. Strains, Plasmid and Materials

The host strain *E. coli* BL21 (DE3) was purchased from WEIDI (Shanghai, China). The L-AI genes from *L. fermentum*, *L. brevis*, *L. plantarum*, and *L. sakei* were all synthesized by Baosai Biotechnology Co., Ltd. (Shanghai, China). Plasmids pET-28a (+), pET-30a (+), and pET-21a (+) were isolated and preserved in our laboratory. Restriction endonucleases, T4 DNA ligase, DNA polymerase, plasmid extraction kits, gel recovery kits, and mutation kits were all purchased from Fujian Herui Biotechnology Co., Ltd. (Xiamen, China).

### 3.2. Construction and Expression of the Recombinant Plasmid

The L-AI gene *araA* from *L. fermentum, L. brevis*, *L. plantarum*, and *L. sakei* was amplified by PCR, yielding the amplified products LFAI-*araA*, LBAI-*araA*, LPAI-*araA*, and LSAI-*araA*. The primers used are listed in Table S2.

LFAI-*araA* and LPAI-*araA*, along with the empty plasmid pET-28a (+), were double-digested with restriction endonucleases EcoR I and Xho I. LBAI-*araA* and the empty plasmid pET-30a (+) were double-digested with EcoR I and BamH I. LSAI-*araA* and pET-21a (+) were double-digested with Nde I and Hind III. Following digestion, the linearized plasmid fragment was recovered using a gel extraction kit. The *araA* gene fragments and the linearized plasmids were then recombined. The recombination reaction mixture was incubated at 37 °C for 30 min. For transformation, 10 μL of the recombinant plasmid was gently mixed with competent *E. coli* BL21 (DE3) cells and placed on ice for 25 min (Table S3). The cells were then heat-shocked in a 42 °C water bath for 45 s and immediately placed back on ice for 2 min. Subsequently, 700 μL of antibiotic-free LB medium was added, and the cells were recovered at 37 °C with shaking at 200 rpm for 60 min. Finally, the cells were collected by centrifugation, plated onto LB agar containing antibiotics, and cultured at 37 °C for 12 h. Recombinant strains *E. coli* BL21(DE3)/pET-28a-LFAI-araA, *E. coli* BL21(DE3)/pET-30a-LBAI-araA, *E. coli* BL21(DE3)/pET-28a-LPAI-araA, and *E. coli* BL21(DE3)/pET-21a-LSAI-araA were successfully constructed.

### 3.3. Induced Expression of L-AI

The recombinant strains were activated by overnight shaking at 37 °C in LB liquid medium containing the corresponding antibiotics (final concentration of 100 μg/mL). Subsequently, they were inoculated at 1% (*v*/*v*) into fresh antibiotic-supplemented LB medium and cultured with shaking at 37 °C and 220 rpm until OD_600_ = 0.6–0.8. After that, IPTG was added to a final concentration of 0.1 mM to induce L-AI expression under conditions of 16 °C and 220 rpm for 24 h.

### 3.4. Single Factor Experiments Optimization of Induction and Reaction Conditions

The four activated recombinant strains were separately transferred into LB medium and fermented under varying induction conditions: different inducer addition timings (4 h, 6 h, 8 h, 10 h, and 12 h), different inducer concentrations (0.1 mM, 0.2 mM, 0.3 mM, 0.4 mM, 0.5 mM, 0.6 mM, 0.7 mM, 0.8 mM, 0.9 mM, and 1.0 mM), and different induction durations (16 h, 20 h, 24 h, 28 h, 32 h, 36 h, and 40 h). The optimal protein expression conditions for each strain were determined based on the conversion rate of D-tagatose.

The induced bacterial cells were collected by centrifugation and resuspended in PBS buffer containing different concentrations of D-galactose (10 g/L, 50 g/L, 100 g/L, 150 g/L, 200 g/L, 250 g/L, and 300 g/L). The reaction was carried out at different temperatures (35 °C, 40 °C, 45 °C, 50 °C, 55 °C, 60 °C, and 65 °C) with shaking at 220 rpm for different durations (12 h, 24 h, 36 h, 48 h, 60 h, and 72 h). The effects of several parameters on production efficiency were investigated to determine the optimal reaction conditions, including initial pH (5.0, 5.5, 6.0, 6.5, 7.0, 7.5, and 8.0), whole-cell concentration (5, 15, 25, 35, 45, 55, 65, 75, 85, 95, and 105 g/L), metal ions Co^2+^ (0, 1, 2, 3, 4, and 5 mM) and metal ion Mn^2+^ (0, 1, 2, 3, 4, and 5 mM).

### 3.5. Site-Directed Mutagenesis and Induced Expression of L-AI

Site-directed mutagenesis was performed to generate active site mutants P99H, K142R, and L465R using the constructed expression plasmid pET-21a-LSAI-*araA* as a template and PCR with site-directed mutagenesis primers (Table S4). The PCR reaction (50 μL) consisted of 5 μL of 10x BeyoAmp^TM^ buffer (with Mg^2+^), 10 μL of dNTP Mix (2.5mM each), 2 μL of forward primer, 2 μL of reverse primer, 2 μL of template plasmid, 1 μL of BeyoAmp^TM^ Extra-long DNA polymerase (Beyotime, Shanghai, China), and 28 μL of distilled water. PCR amplification conditions were as shown in Table S5, with 32 cycles of denaturation to extension step. The products were digested with 1 μL of DPN I at 37 °C, transformed into *E. coli* BL21(DE3) by heat shock and cultured overnight in LB medium containing Amp antibiotics. Mutant strains were obtained: *E. coli* BL21(DE3)/pET-21a-*araA*-P99H (LSAI-*araA*-P99H), *E. coli* BL21(DE3)/pET-21a-*araA*-K142R (LSAI-*araA*-K142R), and *E. coli* BL21(DE3)/pET-21a-*araA*-L465R (LSAI-*araA*-L465R).

The recombinant strain was inoculated at 1% (*v*/*v*) into LB liquid medium containing Amp and cultured with shaking at 37 °C and 220 rpm until the OD_600_ reached 0.6–0.8, indicating the logarithmic growth phase. Then, IPTG was added to a final concentration of 0.5 mM and incubation was conducted at 16 °C and 220 rpm for 32 h to induce LSAI expression, followed by SDS-PAGE electrophoresis.

### 3.6. Analytical Methods

High-performance liquid chromatography (HPLC) was used to determine the content of D-tagatose. Specifically, Agilent 1260 infinity II system (Agilent Technologies, Santa Clara, CA, USA), equipped with rid detector and chromcore sugar-10ca 6 µm, 7.8 × 300 mm column was used. The mobile phase was ultrapure water, the column temperature was 80 °C, and the flow rate was 0.5 mL/min. The temperature of rid detector was set at 35 °C, and the injection volume was 10 μL. Experiments were performed in triplicates of biological replicates and carried out at separate times. The mean of the data is shown in the figures with error bars representing standard deviations.

## 4. Conclusions

Recombinant strains *E. coli* BL21(DE3)/pET-28a-LFAI-araA, *E. coli* BL21(DE3)/pET-30a-LBAI-araA, *E. coli* BL21(DE3)/pET-28a-LPAI-araA, and *E. coli* BL21(DE3)/pET-21a-LSAI-araA were constructed, and the conditions for their catalytic production of D-tagatose were optimized. The results demonstrated that L-AI derived from *L. sakei* exhibited outstanding catalytic performance at a high substrate concentration of 250 g/L, achieving a D-tagatose yield of 36.24% and overcoming the inhibition effect under high substrate concentrations. Bioinformatics analysis of L-AI from *L. sakei* revealed that site-directed mutagenesis can improve the efficiency of the enzyme-catalyzed reaction. Specifically, substituting proline at position 99 with histidine resulted in the highest whole-cell bioconversion performance for D-tagatose production, indicating that the amino acid modification at the 99 site effectively optimized the spatial conformation of the enzyme and its substrate-binding capacity, thereby enhancing whole-cell biotransformation performance under high substrate stress. At a substrate concentration of 250 g/L, the *L. sakei*-araA-P99H mutant achieved a conversion rate of 45.73%, representing a 32.7% increase in catalytic capacity. This result significantly outperforms the catalytic levels of most similar microbial isomerases and effectively addresses the problem of low efficiency at high substrate concentrations in traditional biocatalytic systems. In summary, the above studies show great potential for large-scale D-tagatose synthesis, with the ability to improve tagatose yield and reduce costs. In the future, further advancement toward industrial application can be pursued through fermentation process scale-up, immobilized enzyme engineering, and multi-enzyme system coupling optimization.

## Figures and Tables

**Table 1:** The comparison of substrate concentration and production methods studied on the yield of D-tagatose.

Microorganism	Production Method	Substrate	Conversion Rate	References
*Bacillus* *amyloliquefaciens* CAAI	Enzyme catalysis	100 g/L, D-galactose	47%	[36]
*Bacillus subtilis* WB600-pMA5-LAI	Whole-cell catalysis	90 g/L, D-galactose	43%	[31]
*Lactobacillus parabuchneri* CICC 6004	Whole-cell catalysis	140 g/L, D-galactose	39%	[28]
*Bacillus subtilis* 168 D1/pMA5-alrA-araA-lacZ	Whole-cell catalysis	500 g/L, D-galactose	19%	[32]
*Lactobacillus plantarum* WU14	Whole-cell catalysis	144 g/L, D-galactose	40%	[37]
*Geobacillus stearothermophilus* DSM22	Enzyme catalysis	54 g/L, D-galactose	45%	[38]
tagatose 4-epimerase from *Thermotogae* M4-4	Enzyme catalysis	100 g/L, D-galactose	23%	[39]
		300 g/L, D-galactose	14%	
*Bifidobacterium adolescentis*	Enzyme catalysis	18 g/L, D-galactose	57%	[34]
*Lactobacillus sakei-araA*-P99H	Whole-cell catalysis	10 g/L, D-galactose	75%	This work
		250 g/L, D-galactose	46%	

## Data Availability

The raw data supporting the conclusions of this article will be made available by the authors on request.

## Supplementary Materials

The following supporting information can be downloaded at: https://www.mdpi.com/article/10.3390/molecules31162756/s1.
