# Supplementary material for: Engineering the L-Arabinose Isomerase from Lactobacillus sakei for Whole-Cell Conversion Production of D-Tagatose Under High Substrate Concentration Conditions
Source: Molecules. 2026 Aug 8;31(16):2756. doi: 10.3390/molecules31162756 (PMC13515224; doi:10.3390/molecules31162756)
Supplement: Supplementary file 1 [file molecules-31-02756-s001.zip › molecules-4446614-supplementary.pdf]

Engineering the L-Arabinose Isomerase from *Lactobacillus sakei* for Whole-Cell Conversion Production of D-Tagatose under High Substrate Concentration Conditions

Table S1 Homology alignment of the wild-type LSAI-*araA* sequence.

Rank	PDB ID	Source organism	Sequence identity (%)	E-value	Alignment length	Gap number	Resolution (Å)	Experiment-al method
1	4LQL	*Limosilactobacillus fermentum*	65.82	0	474	0	3.23	X-ray diffraction
2	4R1O	*Geobacillus kaustophilus*	53.91	0	473	0	-	X-ray
3	7CX7	Synthetic construct	53.49	0	473	0	-	X-ray
4	7CHL	Chimeric arabinose isomerase	52.34	2.40E-170	470	3	-	X-ray
5	7CWV	*Thermotoga maritima*	47.59	2.30E-160	475	6	-	X-ray
6	2AJT	*Escherichia coli*	47.11	1.30E-156	467	3	-	X-ray

Table S2 Primer sequences for *araA* gene amplification.

Primer name	primer sequence
LFAI-*araA*-F	CCGGAATTCATGCGTAAGATGCAAGATTACA
LFAI-*araA*-R	CCGCTCGAGCTACTTGATGTTGATAAAGTCG
LBAI-*araA*-F	CGCGGATCCATGTTATCAGTTCCAGATTATG
LBAI-*araA*-R	CCGGAATTCTTACTTGATGAACGCCTTTGTC
LPAI-*araA*-F	CCGGAATTCATGTTATCAGTACCTGATTATG
LPAI-*araA*-R	CCGCTCGAGTTACTTTAAGAATGCCTTAGTC
LSAI-*araA*-F	CGCCATATGATGTTAAATACAGAAAATTATG
LSAI-*araA*-R	CCCAAGCTTTTATTTAATATTGACGTAAGTC

Table S3 Recombinant reaction systems

component	volume
5×CE^{II} Buffer	4 μL
Exnase TM^{II}	2 μL
araA gene segment	0.6 μL
Linearized plasmid fragment	0.9 μL
ddH_2O	12.5 μL
Total	20 μL

Table S4 Mutant primer sequences.

Primer name	primer sequence
araA-P99H-F	CTGCAGAAACATCTGCTGCATCTGGCGACG
araA-P99H-R	ATGCAGCAGATGTTTCTGCAGCAGTTTGGTGC
araA-K142R-F	ACGCGCGCCTGCGCCGCAACAACAAAATTATT
araA-K142R-R	TAATTTTGTTGTTGCGGCGCAGGCGCGCGTT
araA-L465R-F	CTGAGCCATCGCTTTGATCTGACCTAT
araA-L465R-R	GTCAGATCAAAGCGATGGCTCAGATCTTG

Table S5 PCR amplification conditions

	Pre-denaturation	Denaturation	Annealing	Extension	Final Extension
LSAI-P99H	95 ℃<br>3 min	95 ℃<br>20 s	63 ℃<br>30 s	72 ℃<br>6 min	72 ℃<br>10 min
LSAI-K142R	95 ℃<br>3 min	95 ℃<br>20 s	65 ℃<br>30 s	72 ℃<br>6 min	72 ℃<br>10 min
LSAI-L465R	95 ℃<br>3 min	95 ℃<br>20 s	62 ℃<br>30 s	72 ℃<br>6 min	72 ℃<br>10 min

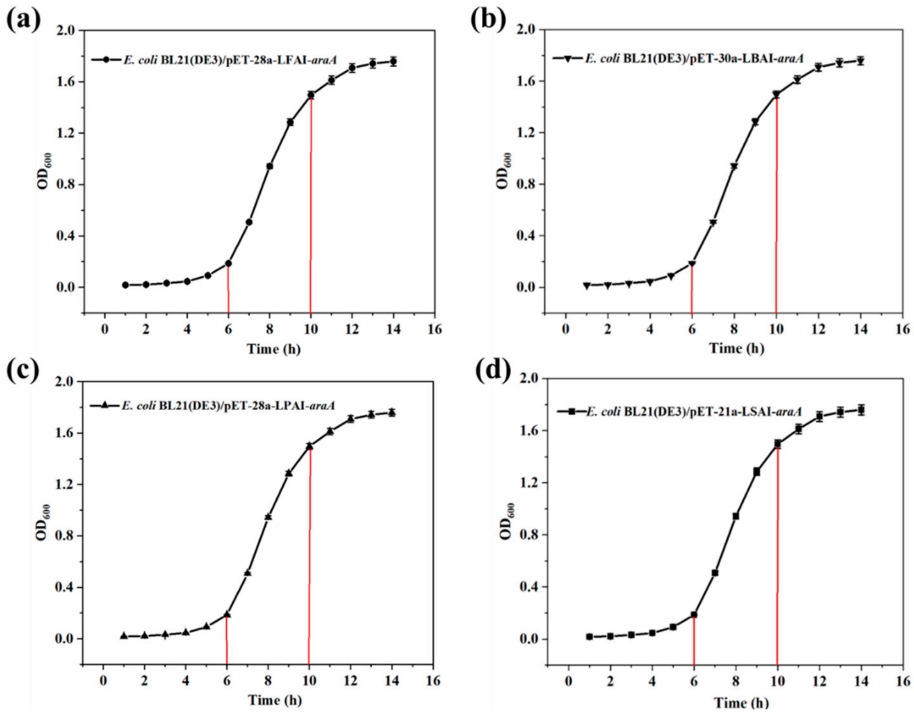

Figure S1 (a) Growth curves of *E. coli* BL21(DE3)/pET-28a-LFAI-*araA*; (b) Growth curves of *E. coli* BL21(DE3)/pET-30a-LBAI-*araA*; (c) Growth curves of *E. coli* BL21(DE3)/pET-28a-LPAI-*araA*; (d) Growth curves of *E. coli* BL21(DE3)/pET-21a-LSAI-*araA*.

(a)

9 Single Mutations generated for L.s_HECHENG

Index	Mutation	Mutation Energy (kcal/mol)	Effect
1	A:PRO99>HIS	-0.69	STABILIZING
2	A:LEU465>HIS	-0.30	NEUTRAL
3	A:ASN144>HIS	-0.13	NEUTRAL
4	A:LEU96>HIS	-0.06	NEUTRAL
5	A:GLN97>HIS	-0.02	NEUTRAL
6	A:LEU100>HIS	0.06	NEUTRAL
7	A:TYR170>HIS	0.20	NEUTRAL
8	A:LYS142>HIS	0.33	NEUTRAL
9	A:ASN143>HIS	3.82	DESTABILIZING

(b)

9 Single Mutations generated for L.s_HECHENG

Index	Mutation	Mutation Energy (kcal/mol)	Effect
1	A:LYS142>ARG	-0.83	STABILIZING
2	A:ASN144>ARG	-0.36	NEUTRAL
3	A:LEU465>ARG	-0.25	NEUTRAL
4	A:PRO99>ARG	-0.19	NEUTRAL
5	A:ASN143>ARG	-0.18	NEUTRAL
6	A:LEU100>ARG	-0.17	NEUTRAL
7	A:LEU96>ARG	-0.13	NEUTRAL
8	A:TYR170>ARG	0.01	NEUTRAL
9	A:GLN97>ARG	0.02	NEUTRAL

(c)

9 Single Mutations generated for L.s_HECHENG

Index	Mutation	Mutation Energy (kcal/mol)	Effect
1	A:LEU465>ARGN	-0.61	STABILIZING
2	A:LYS142>ARGN	-0.49	NEUTRAL
3	A:LEU96>ARGN	-0.23	NEUTRAL
4	A:LEU100>ARGN	-0.22	NEUTRAL
5	A:ASN144>ARGN	-0.19	NEUTRAL
6	A:PRO99>ARGN	-0.15	NEUTRAL
7	A:TYR170>ARGN	-0.03	NEUTRAL
8	A:GLN97>ARGN	0.02	NEUTRAL
9	A:ASN143>ARGN	0.63	DESTABILIZING

Figure S2 Results of single-site mutational scanning of candidate residues: (a) proline 99 to histidine (P99H); (b) lysine 142 to arginine (K142R); (c) leucine 465 to arginine.
